# Can Plasma α-Synuclein Help Us to Differentiate Parkinson’s Disease from Essential Tremor?

**DOI:** 10.5334/tohm.600

**Published:** 2021-05-28

**Authors:** Silvia M. Albillos, Olimpio Montero, Sara Calvo, Berta Solano, José María Trejo, Esther Cubo

**Affiliations:** 1 Area of Biochemistry and Molecular Biology, University of Burgos, Pza. Misael Bañuelos s/n. 09001, Burgos, Spain; 2 Consejo Superior de Investigaciones Científicas (CSIC). Av. Francisco Vallés, 8. 47151 – Boecillo (Valladolid), Spain; 3 Research Unit of the Universitary Hospital of Burgos. Avda. de las Islas Baleares, 3. 09006, Burgos, Spain; 4 Neurology Department of the Universitary Hospital Josep Trueta. Avinguda de França, S/N, 17007 Girona, Spain; 5 Avda. de las Islas Baleares, 3. 09006, Burgos, Avda. de las Islas Baleares, 3. 09006, Burgos, Spain

**Keywords:** Parkinson’s disease, Essential tremor, alpha-synuclein, plasma biomarker, diagnosis

## Abstract

**Background:**

Studies have revealed controversial results regarding the diagnostic accuracy of plasma α-synuclein levels in patients with Parkinson’s disease (PD). This study was aimed to analyze the diagnostic accuracy of plasma α-synuclein in PD versus healthy controls and patients with essential tremor (ET).

**Methods:**

In this cross-sectional study, we included de novo (n = 19) and advanced PD patients [OFF (n = 33), and On (n = 35) states], patients with ET (n = 19), and controls (n = 35). The total plasma α-synuclein levels were determined using an ELISA sandwich method. We performed adjusted multivariate regression analysis to estimate the association of α-synuclein levels with group conditions [controls, ET, and de novo, OFF and ON-PD]. We studied the diagnostic accuracy of plasma α-synuclein using the area under the curve (AUC).

**Results:**

The plasma α-synuclein levels were higher in controls compared to PD and ET (p < 0.0001), discriminating de novo PD from controls (AUC = 0.74, 95% CI 0.60–0.89), with a trend towards in advanced PD (OFF state) from ET (AUC = 0.69, 95% CI 0.53–0.84).

**Conclusions:**

This is the first study examining and comparing plasma α-synuclein levels in ET vs. PD and controls. Preliminary findings suggest that plasma α-synuclein levels might help to discriminate de novo and advanced PD from controls and ET.

## Background

Parkinson’s disease (PD) is well characterized for the pathological presence of α-synuclein (α-syn) aggregates in Lewy bodies. α-syn is considered a promising candidate as a biomarker for diagnosing PD, mainly because some of the non-motor early symptoms, such as constipation, depression, and anosmia, might be related to α-syn inclusions within the related tissues [1]. In non-parkinsonian disorders such as essential tremor (ET), the focal presence of Lewy Bodies in the locus coeruleus has been reported [2].

The majority of studies on levels of different α-syn subtypes, including total, oligomeric, and phosphorylated form in body fluids, have been conducted for the cerebrospinal fluid [3]; however, only a few PD studies have investigated α-syn levels in plasma [4]. The results of that plasma α-syn between PD patients and healthy controls have been controversial [5], and to our knowledge, there is no data on peripheral α-syn levels in ET. This study was therefore aimed to study the discriminative diagnostic accuracy of plasma α-syn in PD versus controls and ET.

## Methods

### Design, Participant characteristics and assessments

This cross-sectional, case-control, pilot, observational study was approved by the Burgos and Soria Health Area Institutional Review Board. Written informed consent was obtained from all subjects. We included a consecutive sample of non-demented patients with PD, ET, and healthy control subjects with a Mini-Mental State Examination [MMSE] ≥ 26) [6,7], followed in a movement disorder clinic at Burgos University Hospital. Patients were diagnosed with idiopathic PD according to the UK PD Society brain bank criteria [8], including *de novo* PD and more advanced PD in both conditions, on the OFF-state (early morning before PD medication and approximately 12 hours after the last dose the night before), and ON-state (1 hour after taking their regular PD medication). The severity of PD motor symptoms was assessed by using the motor subscale of the Unified Parkinson’s Disease Rating Scale (mUPDRS) [9], global burden of non-motor symptoms (NMS) using the NMS Scale for PD (NMSS) [10], and cognitive impairment using the Parkinson’s Disease Cognitive Rating Scale (PDCRS) [11]. The diagnosis of ET was established based on the ET international criteria [12]. *D*e *novo PD* diagnosis was supported by [^123^I]FP-CIT SPECT imaging showing dopamine degeneration. Sociodemographics, bio-specimens, and clinical data collection were obtained in one visit. Control subjects were excluded if they were diagnosed with any other neurological disorders or had a family history of neurodegenerative diseases.

### Determination of α-synuclein

The blood samples were collected into commercially available anticoagulant-treated tubes containing EDTA (BD Vacutainer^TM^). After collection, the tubes were labelled and immediately centrifuged for 15 min at 2000 g in a refrigerated centrifuge Allegra X-30-R (Beckman Coulter Inc.) to remove cells and platelets. The plasma samples were then transferred to Eppendorf tubes in aliquots of 500 μL and stored at –80°C until analysis.

The ELISA method was as follows. In brief, 100 μL of a concentration of 1 μg/ml of anti-human α-syn mouse monoclonal antibody S5566 clone Syn211 (Sigma-Aldrich, Inc, US) was used as capture antibody to coat Microlon® 96 well microplates of half area high binding (Greiner Bio-One, GmbH, Germany) in 50 mM Na_2_CO_3_/NaHCO_3_ coating buffer pH 9.6 overnight at 4°C. After washing the plate four times with PBST (phosphate borate saline buffer with 0.05% Tween 20, pH 7.4), non-specific sites were blocked with 100 μL of blocking buffer at 37°C for 1 h (phosphate borate saline buffer – PBS- pH 7.4 with 2% casein). After another washing step, 100 μl of plasma samples diluted ten times in PBST were added to the wells and analyzed in duplicates. Appropriate standards of recombinant human α-syn expressed in *E. coli* S7820 (Sigma-Aldrich, Inc. US) were prepared in the range of 0.625–300 ng/ml in PBST and included in each plate. A total of twelve calibration points were used in duplicates, counting blanks. The plates were incubated at 37°C for 1.5 h in order to perform the capturing step. A washing step followed in the same conditions as mentioned before. Detection antibody was a polyclonal anti-human α-syn produced in rabbit S3062 (Sigma-Aldrich, Inc. US) using as immunogen a synthetic peptide corresponding to a C-terminus sequence (amino acids 111–132 with a C-terminally added lysine). This sequence has no homology with β and α-synuclein making this polyclonal antibody highly specific to α-syn. Incubation at 37°C for 1 h was done so the polyclonal antibody (100 μL, dilution 1:5000) could bind the α-syn fixed to the monoclonal antibody. After an additional washing step, 100 μL of dilution 1:1000 secondary antibody anti-rabbit-HRP conjugated (horse-radish peroxidase) A6154 (Sigma-Aldrich, Inc. US) was added and left stand in incubation at 37°C for 1 h. A final washing step was performed, and the bound HRP activity was assayed using 100 μL of colorimetric reagent with o-phenilendiamine (OPD) as a substrate during 20 min incubation at 37°C. The enzymatic reaction was stopped by the addition of 50 μL H_2_SO_4_ 2 M, and the plates read at 492 nm within 30 min in a SynergyTM HT plate reader (BioTek Instruments, VT, US).

Concentration estimates of total α-syn in the samples run in duplicates were calculated according to the standard curve obtained in each plate, taking into account the dilution factor. The same lot of standards was used each time in order to minimize inter-assay variability. Calibration curves were included in each ELISA plate and adjusted to a four parametric curve (Eq. 1) using Microsoft Excel with Solver.

Eq. 1 y = \frac{{a - d}}{{1 + {{({\textstyle{x \over c}})}^b}}} + d

where *y* is the OD at 492 nm, *x* the concentration in ng/ml and *a, b, c* and *d* the adjusted parameters.

The method was validated by checking the following parameters. The limit of detection (LOD) was 0.7 ng/mL of α-synuclein. The reproducibility assay showed a sum of squares error for the calibration curves less than 10%. Additionally, the repeatability of the method was measured 20 times with known concentrations of α-synuclein (1.25; 5; 20 and 50 ng/mL) with the coefficient of variation <10% for all samples. The matrix effect was minimized by diluting the plasma ten times prior to analysis, and a standard addition assay (with 0, 10, 15, and 25 ng/mL of α-synuclein) indicated good recoveries in the range of 100 ± 5%.

### Statistical Analysis

The statistics software, IBM SPSS Statistics 19 and Statgraphics Centurion XVII, were used for the analysis of the data. Normal distribution of the variables was analyzed using the Kolmogorov-Smirnov test. Descriptive analysis of the participants’ characteristics was performed in terms of frequencies (percentage), mean/median values with the corresponding standard deviation or interquartile range, as appropriate, and 95% confidence intervals. Based on the median distribution of the age, individuals were classified as younger and older participants. The median values of α-synuclein were compared using the Kruskal-Wallis (several groups), Mood’s and T-Student tests, based on the normal distribution of the data. The pairwise comparisons between groups were performed using the Bonferroni procedure, pointing out statistically significant differences, and Box-and-Whisker plots. The area under the curve (AUC) of the plasma α-syn levels according to the distribution of their concentrations and the diagnosis of PD (*de novo*, OFF-PD, ON-PD) *vs*. ET and controls was used as a measurement of diagnostic accuracy. In this study, we selected an AUC > 0.70 as an indicator of an adequate ability to discriminate between disease conditions [13]. We performed linear multivariate regression analysis to estimate the association of α-synuclein levels with group conditions (ET, *de novo* PD, OFF-PD, ON-PD) with the control group as the reference group, adjusted for age and gender.

## Results

A total of 35 healthy controls, 19 *de novo-*PD, 35 patients with advanced disease evaluated during the ON state (n = 35) and OFF state (n = 33), and 19 ET patients were included. The comparison of clinical characteristics of participants are presented in ***Table 1***. There was a higher proportion of females in the control group than in the other groups (p = 0.01), and patients with ET were older than controls (p = 0.002). Overall, the plasma α-syn levels were weakly, inversely correlated with age (*r_s_* = –0.24, p = 0.004). In PD, the plasma α-syn levels were weakly correlated with the PDCRS (*r_s_* = 0.28, p = 0.01) and moderately, inversely correlated with mUPDRS-III scores (*r_s_* = –0.38, p = 0.03).

**Table 1:** Comparison of participant’s characteristics.


	NOVO-PD	PD		ET	Controls	P VALUE
		
	N=19	ON-STATE N=35	OFF-STATE N=33	N=19	N=35	

Males (%)	13 (68)	24 (69)	22 (67)	12 (63)	11 (31)	0.01

Age (years)*	66.9 (8.8)	64.5 (9.1)	64.1 (9.0)	70.8 (6.7)	61.4 (7.3)	0.002

Disease duration (years)	1 (0.6; 3)	10 (2; 25)	10 (2; 25)	7 (1; 29)	-	-

UPDRS-III score	20 (8; 41)	24 (5; 49)	35 (12; 70)	-	-	0.01

NMSS total score	42 (24; 86)	69 (12; 119)	69 (12; 119)	-	-	0.08

PDCRS total score	74 (58; 112)	80 (43; 109)	69 (12; 119)	-	-	0.91


Values are expressed in medians (interquartile range) otherwise specified *= mean (Standard deviation). Parkinson’s disease (PD) (total sample = 35) measured in the ON state (ON-PD) (n = 35) and OFF state (OFF-PF), (n = 33); ET = Essential Tremor; UPDRS = Unified Parkinson’s disease Rating Scale; NMSS = Non motor symptoms severity score: PDCRS = Parkinson’s disease cognitive rating scale. Data was compared using the ANOVA test (several groups) and the Mann-Whitney U test (2 groups).

**Figure 1:**
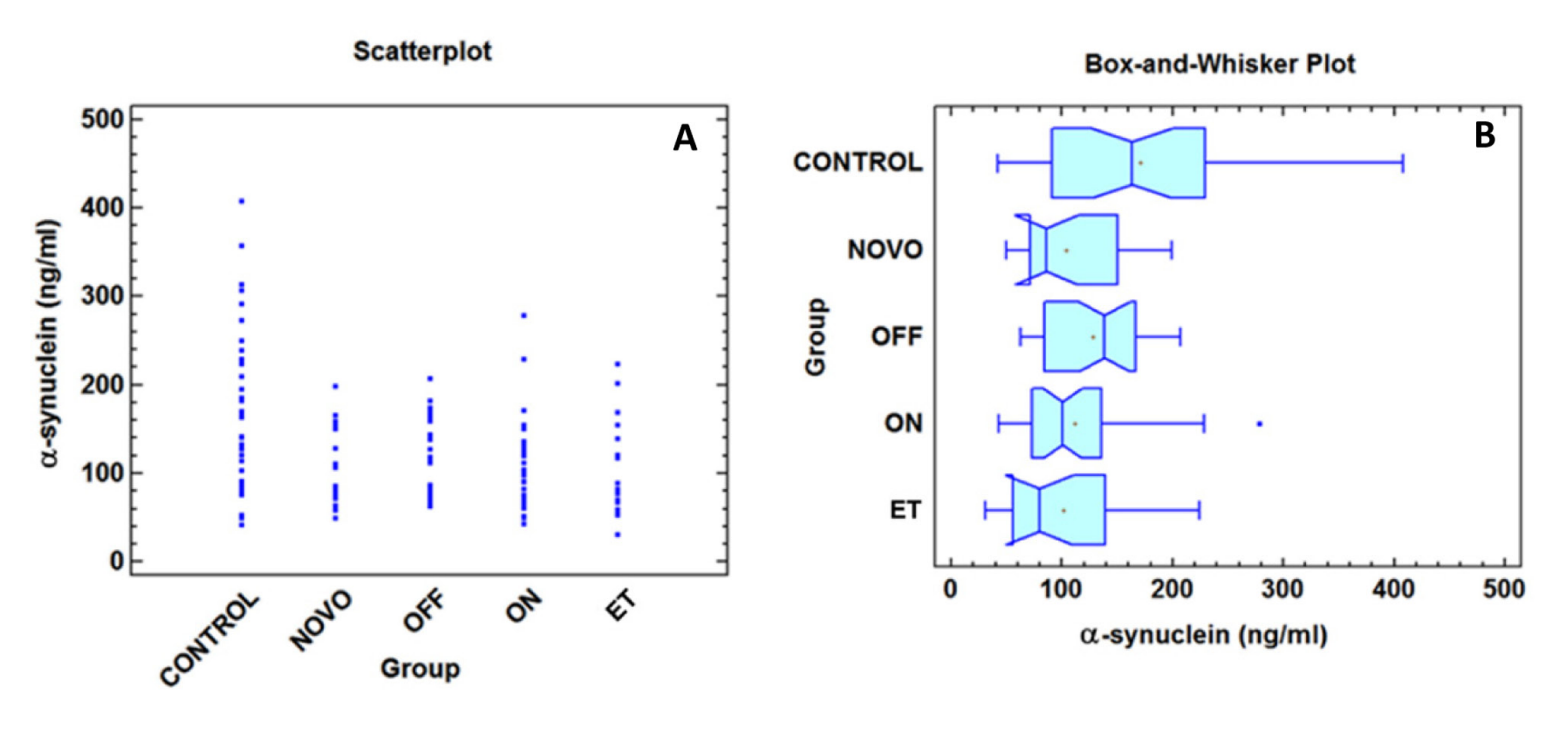
**A)** Scatterplot for the plasma α-synuclein quantitation (ng/ml) for each group. **B)** Box-and-Whisker plot for plasma α-synuclein levels in each group. The median is represented by the vertical line, and a plus sign indicates the mean value. The notch indicates the 95% confidence intervals for the medians. Outside points are also represented. NOVO = *de novo*-PD; OFF = Parkinson’s disease (OFF state); ON = Parkinson’s disease (ON state); ET = Essential Tremor.

The quantitative results for plasma α-syn obtained by means of a sandwich ELISA are shown in ***Figure 1.A***. The median value for the control group was 163.03 ng/ml, which was higher than those of the other groups (*de novo-*PD = 86.11 ng/ml; OFF-PD = 138.45 ng/ml; ON-PD = 100.65 ng/ml and ET = 79.65 ng/ml). The Kruskal-Wallis test indicated that all α-syn mean values were significantly different (p-value = 0.001 at 95% confidence level), with the same result obtained by the Mood’s median test (p-value = 0.01). When the Bonferroni correction was applied to the pairwise comparisons between groups, the α-syn level in the control group was significantly different from *de novo*-PD, ON-PD, and ET groups. Nevertheless, no statistically significant differences were observed between PD (OFF, ON, *de novo-*PD) vs. ET. This can be graphically observed in ***Figure 1.B***, where the Box-and-Whisker plot shows overlapping notches for the control and OFF boxes but non-overlapping notches for the control box compared to the *de novo*, ON-PD, and ET groups. Overall, plasma α-syn levels were similar in terms of age and gender (***Table 2***). However, stratified pairwise comparisons (***Table 2***) showed that in men, plasma α-syn levels were higher in controls than ET, and OFF-PD than *de novo*-PD and ET; in women, plasma α-syn levels were higher in controls than ON and OFF-PD; in young participants (<66 years old) plasma α-syn levels were higher in controls than ON-PD and *de novo*-PD, and OFF-PD than ET. Gender and age-adjusted multivariate regression analysis (***Table 3***), showed that compared to controls, α-syn levels were lower in PD (On, Off and *de novo*-PD) and ET.

**Table 2:** Plasma α-synuclein quantitation (ng/ml) stratified by age and gender.


GROUPS	α-SYNUCLEIN (NG/ML) MEAN ± SD			α-SYNUCLEIN (NG/ML) MEAN ± SD		
			
	MEN (N)	WOMEN (N)	p-VALUE	< 66 YEARS OLD (N)	> 66 YEARS OLD (N)	p-VALUE

CONTROL _(1)_	158.40 ±102.95 (11)	177.12 ±86.53 (24)	0.57	174.81 ±94.85 (25)	162.32 ±84.18 (10)	0.719

ON-PD _(2)_	112.30 ±56.63 (23)	111.08 ±40.45 (10)	0.95	125.76 ±56.77 (17)	97.24 ±42.45 (16)	0.114

OFF-PD _(3)_	128.64 ±46.37 (21)	127.26 ±29.16 (10)	0.92	137.40 ±43.43 (16)	118.37 ±37.40 (15)	0.203

NOVO-PD _(4)_	91.92 ±35.28 (13)	131.25 ±50.01 (6)	0.06	90.21 ±26.38 (7)	112.58 ±49.91 (12)	0.219

ET _(5)_	79.74 ±32.11 (12)	143.73 ±71.89 (6)	0.08	54.33 ±1.95 (2)	106.92 ±56.94 (16)	

	_(1)(5)/ (3)(4)/ (3)(5)_	_(1)(2)/ (1)(3)_	<0.05*	_(1)(2)/ (1)(4)/ (3)(4)_	n.s	<0.05*


SD = Standard deviation. Age was stratified based on the median distribution of the total sample.*Pairwise group comparisons. n.s. = not significant.

**Table 3:** Clinical variables associated with Plasma α-synuclein levels.


N =136 REFERENCE GROUP = CONTROL	B COEF.	IC95%	p-VALUE

ON-PD	–47.83	(–79.33, –16.33)	0.003

OFF-PD	–32.02	(–63.83, –0.20)	0.049

NOVO-PD	–53.50	(–90.57, –16.42)	0.005

ET	–53.28	(–92.84, –13.71)	0.009

Gender (Men)	–22.15	(–45.33, 1.03)	0.06

Age (>=66)	–15.06	(–38.26, 8.15)	0.20

Cons	182.50	(159.17, 205.83)	<0.001


Adjusted *R^2^: 15,1%*.

In ROC analysis, α-syn levels were able to discriminate between 1) *de novo*-PD from controls with an AUC = 0.74, (95% CI 0.60–0.89), and a cut-off α-syn level of 87.65 ng/ml, positive predictive value (PPV) of 0.76 (95% CI 0.59–0.88), negative predictive value (NPV) of 0.62 (95% CI 0.36–0.82); 2) ET from controls with an AUC = 0.74, (95% CI 0.61–0.88), a cut-off α-syn level of 85.80 ng/ml, PPV of 0.73 (95% CI 0.40–0.92), NPV of 0.76 (95% CI 0.60–0.86); with a trend towards discriminating between 1) OFF-PD from controls, with an AUC = 0.64, (95% CI 0.50–0.77), and a cut-off α-syn level of 182.03 ng/ml, 2) OFF-PD from *de novo*-PD, with an AUC = 0.68, (95% CI 0.52–0.83), and a cut-off α-syn level of 111.83 ng/ml; and 3) OFF-PD from ET, with an AUC = 0.69, (95% CI 0.53–0.84), and a cut-off α-syn level of 83.71 ng/ml. α-syn levels did not discriminate *de novo-*PD from ET, and ON-PD from ET and OFF-PD.

## Discussion

The association between ET and PD has frequently been reported [14]. It is well known that a subgroup of patients with ET develops parkinsonian disturbances [15]. On the contrary, action tremor may occur in patients with PD causing misdiagnosis with ET [16,17]. Given the overlapping clinical manifestations and pathology, the differentiation between PD and ET is difficult [18]. Currently, to differentiate PD from ET, we can use [123] I-FP-CIT SPECT [19], or transcranial sonography of the substantia nigra [20], not always available in clinical facilities. Moreover, other procedures such as electrophysiology cannot be used as an accurate diagnostic tool for ET given the wide range of allowable frequency of tremors from 4 to 12 Hz [18]. In terms of fluid biomarkers, to our knowledge, no reliable biomarker for distinguishing PD from ET has been investigated.

In this study, we found that, firstly, in adjusted multivariate analysis, total α-syn levels in plasma were higher in controls compared to PD and ET; secondly, increased α-syn levels in plasma might differentiate advanced PD (OFF state) from ET, and *de novo-*PD, suggesting that capacity of α-syn levels to differentiate PD and ET is influenced by the progression of PD; thirdly, the α-syn levels in plasma did not differentiate ON vs. OFF states, demonstrating that the PD motor state and the time of the medication intake should not be an important confounder; and fourthly, α-syn levels might differentiate *de novo* vs. more advanced PD. Post-hoc analysis (age and gender intra-group comparisons) also demonstrated differences between controls vs. PD and ET. However, our results should be taken with caution, given the lack of adjustment for multiple comparisons due to the overall exploratory approach of the analysis and the absence of comparable preliminary data in ET.

In PD biomarker literature, there is a high heterogeneity across plasma α-syn studies. Whereas some studies have found a reduction of α-syn levels in plasma via ELISA detection for the non-carriers and carriers of mutation LRRK2 [21], other studies have found similar α-syn levels [22], or higher α-syn levels in patients with PD [5] and other parkinsonian disorders such as Multiple System Atrophy compared to controls [23]. These differences across PD studies could be attributed to the co-existence of several components such as assays, disease staging, disease duration, and study setting that may influence the measurement. The discrepancy of results in the literature and the difficulties of interpreting the value of plasma α-syn could be explained by 1) the fact that the peripheral origin of α-syn could be from red cells due to hemolysis [24], and the α-syn content may be overestimated; 2) disparities in methods of analysis, and the sensitivity or specificity of antibodies (total α-syn, oligomeric or modified via phosphorylation α-syn, or even specific isoforms) [25]; 3) the difficulties of controlling the necessary standardization of the protein and its degree of oligomerization [3]; 4) the interference of heterophilic antibodies present in up to 40% of the population, which have affinity for animal antibodies [26], and oligomeric detection due to the competition of monomers if the same antibody is used for capture and detection, reducing the number of oligomers quantified [27]; and 5) the interference of endogenous peroxidases and alkaline phosphatases present in blood components, as these endogenous enzymes may increase the assay background. To overcome methodological bias in determining the value of plasma α-syn, our methodology was in-house, carefully described, and the measurements were all performed in duplicates and validated.

Our study has some limitations, including the small sample size of patients and a cross-sectional design. On the other hand, our results were obtained from a single-center, and the biosamples were standardly collected and analyzed in all participants, decreasing collection and measurement bias. In addition, our study can be useful for prompting further studies with larger sample cohorts that will allow to determine whether α-syn levels in plasma have a diagnostic capacity to discriminate between predominant tremor phenotype of PD from ET. We have also highlighted the importance of providing detailed information on *how* and *when* the plasma α-syn is collected, in terms of PD severity (*de novo* vs. advanced PD), and time (OFF vs. ON state), in order to establish standard protocols.

In sum, the current study provides evidence in support of the possibility that α-syn levels in plasma might help to differentiate PD from controls and advanced PD from ET. There is no doubt that for biomarker development applied to large tremor populations, the field should evolve into an era of etiologically defined tremor disorders.

## References

[1] Delenclos M, Jones DR, McLean PJ, Uitti RJ. Biomarkers in Parkinson’s disease: Advances and strategies. Parkinsonism Relat Disord. 2016; 22 Suppl 1: S106–110. DOI: 10.1016/j.parkreldis.2015.09.048 PMC5120398 26439946

[2] Louis ED, Faust PL, Vonsattel JP, et al. Neuropathological changes in essential tremor: 33 cases compared with 21 controls. Brain. 2007; 130: 3297–3307. DOI: 10.1093/brain/awm266 18025031

[3] Mollenhauer B, Caspell-Garcia CJ, Coffey CS, et al. Longitudinal CSF biomarkers in patients with early Parkinson disease and healthy controls. Neurology, 2017; 89: 1959–1969. DOI: 10.1212/WNL.0000000000004609 29030452 PMC5679418

[4] Gupta V, Garg RK, Khattri S. Serological Analysis of Alpha-synuclein and NF-kappaB in Parkinson’s Disease Patients. J Clin Diagn Res. 2015; 9: BC01–04. DOI: 10.7860/JCDR/2015/12545.5978 PMC4484062 26155470

[5] Bougea A, Stefanis L, Paraskevas GP, Emmanouilidou E, Vekrelis K, Kapaki E. Plasma alpha-synuclein levels in patients with Parkinson’s disease: a systematic review and meta-analysis. Neurological sciences: official journal of the Italian Neurological Society and of the Italian Society of Clinical Neurophysiology. 2019; 40: 929–938. DOI: 10.1007/s10072-019-03738-1 30715632

[6] Folstein MF, Folstein SE, McHugh PR. “Mini-mental state”. A practical method for grading the cognitive state of patients for the clinician. J Psychiatr Res. 1975; 12: 189–198. DOI: 10.1016/0022-3956(75)90026-6 1202204

[7] Dubois B, Burn D, Goetz C, et al. Diagnostic procedures for Parkinson’s disease dementia: recommendations from the movement disorder society task force. Mov Disord. 2007; 22: 2314–2324. DOI: 10.1002/mds.21844 18098298

[8] Hughes AJ, Daniel SE, Kilford L, Lees AJ. Accuracy of clinical diagnosis of idiopathic Parkinson’s disease: A clinico-pathological study of 100 cases. J Neurol Neurosurg Psychiatry. 1992; 55: 181–184. DOI: 10.1136/jnnp.55.3.181 1564476 PMC1014720

[9] Fahn S, Elton R. Members of the UPDRS Development Committee Recent Developments in Parkinson’s Disease, Vol 2. Florham Park, NJ: Macmillan Health Care Information; 1987.

[10] Chaudhuri KR, Martinez-Martin P, Brown RG, et al. The metric properties of a novel non-motor symptoms scale for Parkinson’s disease: Results from an international pilot study. Mov Disord. 2007; 22: 1901–1911. DOI: 10.1002/mds.21596 17674410

[11] Pagonabarraga J, Kulisevsky J, Llebaria G, Garcia-Sanchez C, Pascual-Sedano B, Gironell A. Parkinson’s disease-cognitive rating scale: A new cognitive scale specific for Parkinson’s disease. Mov Disord. 2008; 23: 998–1005. DOI: 10.1002/mds.22007 18381647

[12] Bain P, Brin M, Deuschl G, et al. Criteria for the diagnosis of essential tremor. Neurology, 2000; 54: S7. 10854345

[13] Tape TG. Interpreting diagnostic tests. University of Nebraska Medical Center, 2008. (Accessed September 10, 2020, at http://gim.unmc.edu/dxtests/Default.htm).

[14] Louis ED, Ottman R. Is there a one-way street from essential tremor to Parkinson’s disease? Possible biological ramifications. European journal of neurology. 2013; 20: 1440–1444. DOI: 10.1111/ene.12256 24033795 PMC3801177

[15] Minen MT, Louis ED. Emergence of Parkinson’s disease in essential tremor: A study of the clinical correlates in 53 patients. Mov Disord. 2008; 23: 1602–1605. DOI: 10.1002/mds.22161 18618664 PMC2683412

[16] Tarakad A, Jankovic J. Essential Tremor and Parkinson’s Disease: Exploring the Relationship. Tremor and other hyperkinetic movements (New York, NY). 2018; 8: 589. DOI: 10.5334/tohm.441 PMC6329774 30643667

[17] Shahed J, Jankovic J. Exploring the relationship between essential tremor and Parkinson’s disease. Parkinsonism Relat Disord. 2007; 13: 67–76. DOI: 10.1016/j.parkreldis.2006.05.033 16887374

[18] Espay AJ, Lang AE, Erro R, et al. Essential pitfalls in “essential” tremor. Mov Disord. 2017; 32: 325–331. DOI: 10.1002/mds.26919 28116753 PMC5359065

[19] Budisic M, Trkanjec Z, Bosnjak J, Lovrencic-Huzjan A, Vukovic V, Demarin V. Distinguishing Parkinson’s disease and essential tremor with transcranial sonography. Acta Neurol Scand. 2009; 119: 17–21. DOI: 10.1111/j.1600-0404.2008.01056.x 18549415

[20] Yilmaz R, Berg D. Transcranial B-Mode Sonography in Movement Disorders. Int Rev Neurobiol. 2018; 143: 179–212. DOI: 10.1016/bs.irn.2018.10.008 30473195

[21] Gorostidi A, Bergareche A, Ruiz-Martinez J, et al. Alphalpha-synuclein levels in blood plasma from LRRK2 mutation carriers. PLoS One. 2012; 7: e52312. DOI: 10.1371/journal.pone.0052312 23300640 PMC3531490

[22] Lee PH, Lee G, Park HJ, Bang OY, Joo IS, Huh K. The plasma alpha-synuclein levels in patients with Parkinson’s disease and multiple system atrophy. Journal of neural transmission (Vienna, Austria : 1996). 2006; 113: 1435–1439. DOI: 10.1007/s00702-005-0427-9 16465458

[23] Yang F, Li WJ, Huang XS. Alpha-synuclein levels in patients with multiple system atrophy: A meta-analysis. Int J Neurosci. 2018; 128: 477–486. DOI: 10.1080/00207454.2017.1394851 29053035

[24] Barbour R, Kling K, Anderson JP, et al. Red blood cells are the major source of alpha-synuclein in blood. Neurodegener Dis. 2008; 5: 55–59. DOI: 10.1159/000112832 18182779

[25] Shi M, Zabetian CP, Hancock AM, et al. Significance and confounders of peripheral DJ-1 and alpha-synuclein in Parkinson’s disease. Neurosci Lett. 2010; 480: 78–82. DOI: 10.1016/j.neulet.2010.06.009 20540987 PMC2943649

[26] Bolstad N, Warren DJ, Nustad K. Heterophilic antibody interference in immunometric assays. Best Pract Res Clin Endocrinol Metab. 2013; 27: 647–661. DOI: 10.1016/j.beem.2013.05.011 24094636

[27] Bidinosti M, Shimshek DR, Mollenhauer B, et al. Novel one-step immunoassays to quantify alpha-synuclein: applications for biomarker development and high-throughput screening. J Biol Chem. 2012; 287: 33691–33705. DOI: 10.1074/jbc.M112.379792 22843695 PMC3460466

