# Correction: Can Plasma α-Synuclein Help Us to Differentiate Parkinson’s Disease from Essential Tremor?

**DOI:** 10.5334/tohm.637

**Published:** 2021-06-25

**Authors:** Silvia M. Albillos, Olimpio Montero, Sara Calvo, Berta Solano, José María Trejo, Esther Cubo

**Affiliations:** 1 Area of Biochemistry and Molecular Biology, University of Burgos, Pza. Misael Bañuelos s/n. 09001, Burgos, ES; 2 Consejo Superior de Investigaciones Científicas (CSIC). Av. Francisco Vallés, 8. 47151, Boecillo (Valladolid), ES; 3 Research Unit of the Universitary Hospital of Burgos. Avda. de las Islas Baleares, 3. 09006, Burgos, ES; 4 Neurology Department of the Universitary Hospital Josep Trueta. Avinguda de França, S/N, 17007 Girona, ES; 5 Avda. de las Islas Baleares, 3. 09006, Burgos, Avda. de las Islas Baleares, 3. 09006, Burgos, ES

**Keywords:** Parkinson’s disease, Essential tremor, alpha-synuclein, plasma biomarker, diagnosis

## Abstract

This article details a correction to: Albillos SM, Montero O, Calvo S, Solano B, Trejo JM, Cubo E. Can Plasma α-Synuclein Help Us to Differentiate Parkinson’s Disease from Essential Tremor? Tremor and Other Hyperkinetic Movements. 2021; 11(1): 20, pp. 1–8. DOI: *https://doi.org/10.5334/tohm.600*

## Correction

In ***Table 1*** of Albillos et al. [1] there is no Off-State N = 33 value of NMSS total score and of PDCRS total score.

**Table 1:** Comparison of participant’s characteristics.


	NOVO-PD N = 19	PD N = 35		ET N = 19	CONTROLS N = 35	P-VALUE

		ON-STATE	OFF-STATE			

Males (%)	13 (68)	24 (69)	22 (67)	12 (63)	11 (31)	0.01

Age (years)^a^	66.9 (8.8)	64.5 (9.1)	64.1 (9.0)	70.8 (6.7)	61.4 (7.3)	0.002

Disease duration (years)	1 (0.6; 3)	10 (2; 25)	–	7 (1; 29)	–	–

UPDRS-III score	20 (8; 41)	24 (5; 49)	35 (12; 70)	–	–	0.01

NMSS total score	42 (24; 86)	69 (12; 119)	–	–	–	0.08

PDCRS total score	74 (58; 112)	80 (43; 109)	–	–	–	0.91


Values are expressed in medians (interquartile range) otherwise specified ^a^ = mean (Standard deviation). Parkinson’s disease (PD) (total sample = 35) measured in the ON state (ON-PD) (n = 35) and OFF state (OFF-PF), (n = 33); ET = Essential Tremor; UPDRS = Unified Parkinson’s disease Rating Scale; NMSS = Non motor symptoms severity score: PDCRS = Parkinson’s disease cognitive rating scale. Data were compared using the ANOVA test.

In ***Table 2***, the values of α-Synuclein are quoted in ng/ml. The label of the first cell of the last row is ‘Pairwise comparisons’ and should read (1) vs. (5), (2) vs. (4), and (3) vs. (5) (second cell), (1) vs. (2) and (1) vs. (3) (third cell) and (1) vs. (2), (1) vs. (4), and (3) vs. (4) (fifth cell).

**Table 2:** Plasma α-synuclein quantitation (ng/ml) stratified by age and gender.


GROUPS	α-SYNUCLEIN (ng/ml) MEAN ± SD		p-VALUE	α-SYNUCLEIN (ng/ml) MEAN ± SD		P-VALUE
	
	MEN (n)	WOMEN (n)		<66 YEARS OLD (n)	≥66 YEARS OLD (n)	

CONTROL _(1)_	158.40 ± 102.95 (11)	177.12 ± 86.53 (24)	0.57	174.81 ± 94.85 (25)	162.32 ± 84.18 (10)	0.71

ON-PD _(2)_	112.30 ± 56.63 (23)	111.08 ± 40.45 (10)	0.95	125.76 ± 56.77 (17)	97.24 ± 42.45 (16)	0.11

OFF-PD _(3)_	128.64 ± 46.37 (21)	127.26 ± 29.16 (10)	0.92	137.40 ± 43.43 (16)	118.37 ± 37.40 (15)	0.20

NOVO-PD _(4)_	91.92 ± 35.28 (13)	131.25 ± 50.01 (6)	0.06	90.21 ± 26.38 (7)	112.58 ± 49.91 (12)	0.21

ET _(5)_	79.74 ± 32.11 (12)	143.73 ± 71.89 (6)	0.08	54.33 ± 1.95 (2)	106.92 ± 56.94 (16)	

Pairwise comparisons	(1) vs. (5)(2) vs. (4)(3) vs. (5)	(1) vs. (2)(1) vs. (3)	<0.05*	(1) vs. (2)(1) vs. (4)(3) vs. (4)	n.s	<0.05*


SD = Standard deviation. Age was stratified based on the median distribution of the total sample. * Pairwise intra-group comparisons as indicated in each column; n.s = not significant.

The correct tables are included here.

This information was not included correctly in the final versions sent by the authors.
